# Medical Simulation Anywhere and Anytime: Simulation in a Backpack

**DOI:** 10.21980/J8Z94W

**Published:** 2025-01-31

**Authors:** Janice Shin-Kim, Adam Blumenberg

**Affiliations:** * Columbia University Vagelos College of Physicians and Surgeons, Department of Emergency Medicine, New York, NY

## Abstract

**Audience:**

*Simulation in a Backpack* is designed to offer immersive simulation experiences to medical professionals, including emergency medicine and pediatrics residents, medical students, pharmacists, and pharmacy students. It particularly targets those in environments where traditional simulation equipment is not readily accessible.

**Introduction:**

Simulation education plays a crucial role in emergency medicine training, offering hands-on experiences in managing critical resuscitations. *Simulation in a Backpack* was developed to provide portable and quickly set-up simulation sessions, offering a solution for environments lacking simulation laboratories.

**Educational Objectives:**

By the end of this simulation experience learners will be able to:

1. Recognize and manage emergencies through immersive simulation experiences with an inflatable manikin and pre-programmed free software system.
2. Demonstrate knowledge and skills to resuscitate patients with medical emergencies.
3. Enhance confidence and competence in responding to medical emergencies using portable, low-tech resources.
4. Foster interdisciplinary collaboration and effective communication during scenarios.

**Educational Methods:**

*Simulation in a Backpack* utilizes an inflatable manikin and free simulation software, Med Sim Studio, with easy portability and speedy setup, facilitating hands-on learning in realistic scenarios and optional electronic modulators to augment fidelity. The curriculum employs experiential learning theory, engaging learners in reflective experiences and practical application.

**Research Methods:**

The efficacy of *Simulation a Backpack* was evaluated through learner (resident physicians, medical students, pharmacists) feedback. The Net Promoter Score (NPS) and focused qualitative analysis of written feedback were used to assess satisfaction and learning experiences.

**Results:**

Out of 191 learners, *Simulation in a Backpack* received a commendable NPS of +81.33, indicating high satisfaction. Focused qualitative analysis showed positive feedback, with learners valuing the educational experience despite the low-fidelity of the manikin.

**Discussion:**

*Simulation in a Backpack* effectively provided meaningful educational experiences, as evidenced by high NPS and positive written feedback. Modifications were made based on practical experience and learner feedback, emphasizing efficient and immersive experiences and managing expectations regarding fidelity.

This innovation addresses the growing need for portable simulation solutions, offering a cost-effective and flexible approach to simulation in medical training.

**Topics:**

Portable simulation, innovative simulation, interdisciplinary collaboration.

## USER GUIDE

**Table:** 

List of Resources:	
Abstract	1
User Guide	3

**Learner Audience:**

Medical Students, Interns, Junior Residents, Senior Residents, Physician Assistants

**Time Required for Implementation:**

*Simulation in a Backpack* is intended to be a simple, portable solution to simulation education outside a simulation laboratory. To this end, the materials are lightweight, portable, relatively inexpensive, and easily set up for a simulation session anywhere or anytime. Prior to starting a lesson, it takes approximately 20 minutes to inflate the manikin, set up the props, and open the simulation software. Similarly, it takes less than 20 minutes to deflate the manikin and repackage all the materials.

This innovation describes a suite of optional electronic modules that may increase the fidelity of the manikin by providing the capacity to simulate seizures, hypoxia, pulse assessments, bleeding, vomiting, and sound effects. If the instructor chooses to use the optional electronic modules, the initial fabrication process may take about 4–8 hours.

The learners will spend the same amount of time using *Simulation in a Backpack* as any other medical simulation. To account for prebriefing, case presentation, and debriefing, 45–60 minutes should be allocated per case.

**Recommended Number of Learners per Instructor:**

There should be four to eight active learners per simulation session. Additional learners may be present as observers.

**Topics:**

Portable simulation, innovative simulation, interdisciplinary collaboration.

**Objectives:**

1. Recognize and manage emergencies through immersive simulation experiences with an inflatable manikin and pre-programmed free software system.
2. Demonstrate knowledge and skills to resuscitate patients with medical emergencies.
3. Enhance confidence and competence in responding to medical emergencies using portable, low-tech resources.
4. Foster interdisciplinary collaboration and effective communication during scenarios.

### Linked objectives, methods and results

The goals and objectives of the curriculum are achieved by leveraging immersive simulation experiences through the easy portability of an inflatable manikin and free, accessible simulation computer software. The conceptual framework underpinning this curriculum is experiential learning theory, which posits that learning is effective when individuals actively engage in experiences, reflect on those experiences, and apply their learning to future situations.^1^ Using a portable low-fidelity manikin for simulation facilitates learning and skill development in emergencies by engaging learners in realistic hands-on scenarios. It also provides opportunities for reflection and feedback through debriefing and application of knowledge. *Simulation in a Backpack* allows instructors to teach the development and refinement of learners’ resuscitation skills in patient assessment, airway management, and other critical interventions necessary for emergency scenarios.^2^,^3^

### Recommended pre-reading for instructor

If the instructor is a novice simulationist, they may benefit from reading about various prebriefing and debriefing techniques, simulation operations, and simulation software. A focused reading list includes:

*Review of debriefing methods:*

- Sawyer T, Eppich W, Brett-Fleegler M, Grant V, Cheng A. More than one way to debrief: A critical review of healthcare simulation debriefing methods. *Simul Healthc*. 2016;11(3):209–217. doi:10.1097/SIH.0000000000000148

*Simulation as a teaching modality:*

- Lateef F. Simulation-based learning: Just like the real thing. *J Emerg Trauma Shock*. 2010;3(4):348–352. doi:10.4103/0974-2700.70743
- Blumenberg A. *Med Sim Studio software*: Med Sim Studio: An open-access simulation platform for running and sharing dynamic display of patient data in package scenarios by Blumenberg and Kessler 2022.^6^ https://www.medsimstudio.com

### Implementation Methods

To implement the curriculum objectives, the instructional sessions will be designed to maximize engagement and hands-on learning using the innovative and portable *Simulation in a Backpack*. Facilitators will be able to set up the inflatable manikin and pre-programmed simulation software in a PC laptop in 20 minutes.

Each session will start with short prebriefing to remind learners of objectives and orient them to logistics, such as the duration of simulation and what can or cannot be done in simulation. Learners will then participate in immersive simulation exercises where they will encounter realistic scenarios utilizing the inflatable manikin to mimic patient presentations. Throughout scenarios, learners will collaborate and apply their knowledge to assess and manage critically ill patients. The simulation will be followed by debriefing during which facilitators will provide real-time feedback on teamwork and communication and discuss evidence-based management relevant to emergencies and resuscitation skills.

By integrating the portability of an inflatable manikin and freely accessible simulation software, *Med Sim Studio* (www.MedSimStudio.com), facilitators can provide engaging simulation experiences to various learners with flexible locations and resources.

### List of items required to replicate this innovation

#### Manikin

1. Inflatable manikin. Internet search term: “inflatable dress display mannequin.” Estimated price: $30.
2. Air pump. Internet search term: “foot air pump.” Estimated price: $20.
3. Latex facemask. Internet search term: “realistic latex face mask.” Estimated price: $30.
4. Clothing, such as t-shirt and shorts. Estimated price: Variable.
5. Backpack or duffel bag. Estimated price: Variable.
6. Medical equipment as stage props, eg, laryngoscope, syringe, medication vial, etc. Estimated price: Variable; recommend using expired unused materials.
7. (Optional) Packing cubes. Estimated price: $20.

#### Computer Software & Hardware

1. Recommended software: *Med Sim Studio* may be downloaded from www.MedSimStudio.com. Estimated price: Free.
2. Alternative software: *Vital Sign Simulator* may be downloaded from https://sourceforge.net/projects/vitalsignsim/. Estimated price: Free.
3. A laptop computer that operates Microsoft Windows 10 or later. Ensure that there is an HDMI port to support an auxiliary display. Internet search term: “refurbished Windows laptop.” Estimated price: $200–$300.
4. An auxiliary display or video projector. For a truly mobile platform, this can be a small battery-powered monitor. Internet search term: “11.6-inch built-in battery-powered monitor.” Estimated price: $100–$200.

#### Electronic Modules (optional)

1. *Simulating seizures*: Two 6 volt (v) direct current (DC) rumbling motors with small hard-shelled cases, such as a film cannister. Internet search term: “6v DC rumble motor.” Estimated price: $20.
2. *Simulating cyanosis*: Five Blue 5v DC LEDs and thermoplastic beads. Ensure the LEDs are meant to be powered with a power supply that provides 5–6 volts. Internet search terms: “pre-wired 5v blue led” and “thermoplastic beads.” Estimated price: $12.
3. *Pulse Generator*: A square waveform generator. Internet search term “square wave signal generator PCB.” Estimated price: $5–$10.
4. *Simulating vomiting, pulsatile bleeding, or a palpable pulse*: Low-voltage water pump, 1-liter hydration bladder, and non-compressible air brake tubing with connectors. Internet search terms: “6v brushless water pump” (ensure that there are ports which can connect to tubing), “1-liter hydration bladder,” and “8mm polyurethane pneumatic tubing kit.” Estimated price: $30–$40.
5. *Wire connectors* (or soldering equipment if so skilled). Internet search term “quick splice wire connectors.” Estimated price: $10.
6. *A USB power supply capable of delivering at least 10 watts*. Note: some USB batteries automatically shut off after a few minutes if not being used which may interfere with the simulationist’s control. A USB power supply that uses AA batteries or has an “always on” setting should be used. Internet search term: “AA USB battery pack.” Estimated price: $10.
7. *Plastic containers for the various modules*. Estimated price: $20.

#### Electronic Module Simple Controller (optional)

1. Four USB cables with an on/off switch. Internet search term: “USB on off switch cable.” Estimated price: $8.
2. One USB, one-male-to-four-female splitter cable. Internet search term: “USB splitter Y-cable, male to 4 female USB-A expander hub.” Estimated price: $15.

#### Electronic Module Remote Controller (optional)

1. A 4-channel radio-frequency relay. Internet search term: “12-volt 4 channel 433mhz RF relay.” Estimated price: $20.
2. Six Type A USB extension cables, 4–6 feet (120–183cm). Internet search term: “USB type A extension cable 6 pack.” Estimated price: $20.
3. One DC-to-DC step up converter. Internet search term: “5v to 12v step up converter.” Estimated price: $8.

### Approximate cost of items to create this innovation

The cost in US dollars of this innovation may be as low as $75 or as high as $500, depending on what materials the instructor already has and whether the instructor wishes to use the electronic modules. If the instructor already has access to a laptop with Microsoft Windows with an auxiliary display and does not wish to use the optional electronic modules, the cost may be as low as $75. The cost breakdown is approximately $75 for the simulation manikin, $200–$300 for a computer, $100–$200 for an auxiliary display, and $100 for the optional electronic modules. Since most educators already have access to a PC laptop, the projected total cost for *Simulation in a Backpack* is approximately $300.

### Detailed methods to construct this innovation

*Simulation in a Backpack* is fundamentally an innovation wherein an easily portable life-sized manikin, along with medical stimuli, becomes accessible to learners anywhere and anytime. An effective setup is simply bringing a backpack with the manikin, a PC laptop computer with an auxiliary display, and medical props in a backpack.

This innovation may be operated with or without the electronic modules. The electronic modules add additional simulation fidelity and provide the simulationist with a means to communicate clinical findings non-verbally. However, the electronic modules are not required for an effective educational session and should be considered optional.

### Simulation in a Backpack

**Figure 1:**
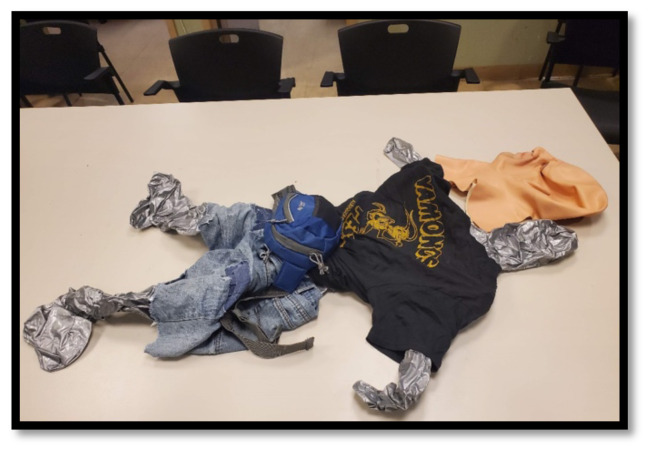
Deflated manikin with clothing and latex mask.

**Figure 2:**
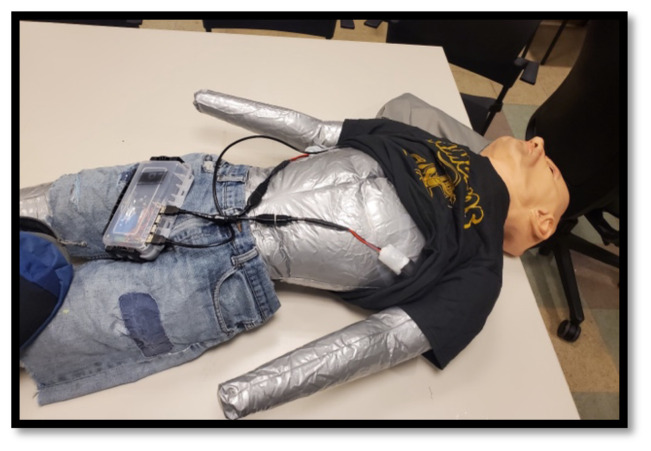
The electronic modules are connected to the controller. Wires are concealed underneath clothing. The controller may be placed in a fanny pack on the manikin’s waist or adjacent to the manikin.

**Figure 3:**
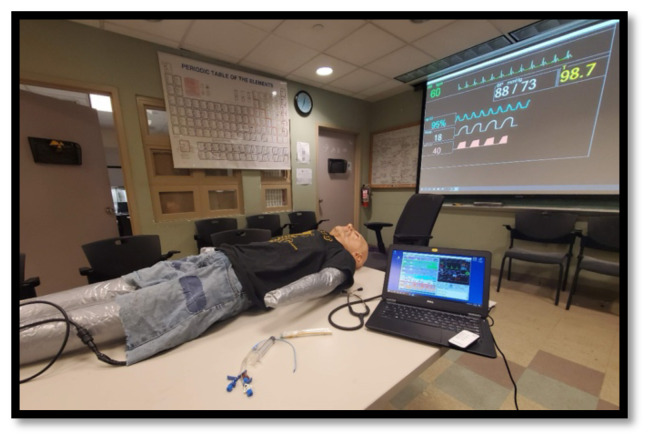
Manikin is placed on a conference room table. Medical props (syringe, central venous catheter, etc.) are adjacent. A PC laptop running Med Sim Studio is connected to a nearby projector to display vital signs and medical images to the learners.

**Figure 4:**
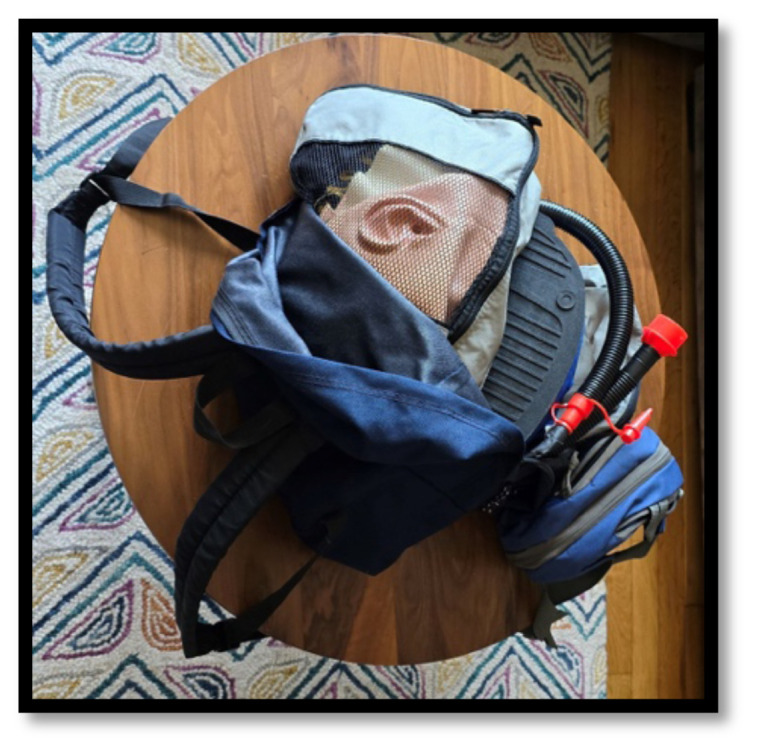
*Simulation in a Backpack:* All the required materials, including manikin, medical props, laptop, auxiliary monitor, air pump, and optional electronic modules, fit inside an easily portable carrying case, such as a backpack.

1. Store the deflated manikin in a backpack with a laptop and medical equipment (Figure 1).
2. Clothe, inflate, and position the manikin prior to a teaching session (Figure 2).
3. Run *Med Sim Studio* on the computer and auxiliary display and begin the education session (Figure 3).
4. After the education session is concluded, deflate the manikin and pack the equipment into the backpack (Figure 4).

### Optional electronic modules

**Figure 5:**
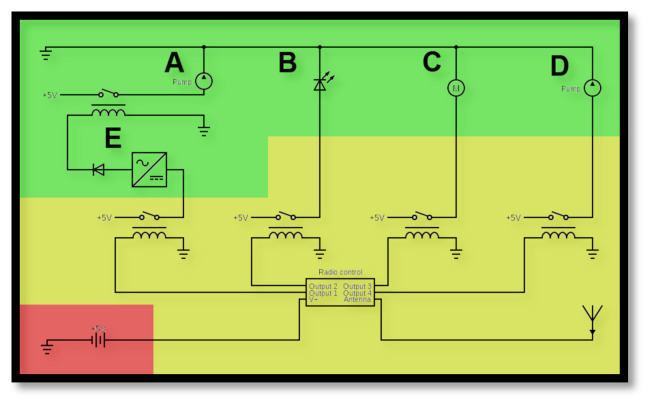
A circuit diagram representing the optional electronic modules connected to the controller. The green represents the modules: A) a water pump controlled by a E) square wave generator and relay for pulsatile flow, B) a blue LED for cyanosis, C) a motor for seizures, and D) a water pump for vomiting. The yellow area of the diagram represents the controller, which is a four-channel relay system controlled by radio-frequency remote. The power supply is in the red area.

The principle of the electronic modules is that the simulationist may enable or disable a clinical finding with the press of a single button. Both the “Simple Controller” and the “Remote Controller” utilize a 5v USB power supply with on/off switches to individually connected modules (Figure 5).

### Simple Controller (Figure 6A)

1. Plug the one-male-to-four-female splitter cable into the USB power supply. Each of the four outputs may be individually controlled by a USB on/off switch.
2. Plug the desired module into the on/off switch.

### Remote Controller (Figure 6B)

1. Connect the DC-to-DC step up converter to the male USB cable and adjust to 12v output using a voltmeter. Connect the positive and negative terminals of the 12-volt 4 channel 433mhz RF relay to the 12v output of the DC-to-DC step up converter.
2. Connect the negative terminals of 4 female USB cables to the negative terminal of the male USB cable.
3. Connect the input of the 4 relays to the positive terminal of the male USB cable. NOTE: Do not connect these to the 12v output from the DC-to-DC step up converter; otherwise, it may damage the downstream electronic modules.
4. Connect the outputs of the 4 relays to the positive terminals of the 4 female USB cables.
5. Place the electronics in a container.

### Pulse module

This module generates a pulsatile on/off signal. It is intended to control a second module, such as a fluid pump or motor.

1. Connect the positive and negative terminals of the square wave generator to the male USB cable. Connect the positive and negative terminals of the relay to the male USB cable. Connect the negative terminal of the female USB cable to the negative terminal of the male USB cable.
2. Connect the positive terminal of the female USB cable to the output of the relay. Connect the relay’s control input to the output signal of the square waveform generator.
3. Adjust the frequency of the square waveform generator to 60–100 cycles per minute.
4. Place the electronics in a plastic electronics enclosure.
5. Place the module in series between the power source and another module, such as the seizure simulator or pulsatile bleeding simulator.

### Cyanosis module (Figure 7)

1. Connect one or more blue LEDs to the positive and negative terminals of the male USB cable.
2. Secure the electronics with thermoplastic or other moldable material.

### Seizure module (Figure 8)

1. Connect the 6v DC rumbling motors to the positive and negative terminals of the male USB cables.
2. Place the electronics in a plastic container. Secure the body of the vibrating motors to the sides of the container so that the motor does not move relative to the container.

### Water pump module (Figure 9)

1. Connect the positive and negative terminals of the water pump to a male USB cable.
2. Connect the water intake port of the water pump to a 1-liter soft hydration pack.
3. Connect the water output port of the water pump to plastic non-compressible brake tubing.
4. Connect the distal end of the brake tubing to compressible latex tubing.
  - For a palpable pulse, clamp the distal end of the latex tubing. Plug the water pump module into the pulse module. Fill the hydration bladder with water.
  - For pulsatile bleeding, clamp the distal end of the latex tubing, and make a 1cm hole into the middle of the latex tubing. Plug the water pump module into the pulse module. Fill the hydration bladder with fake blood.
  - For vomiting, leave the distal end of the latex tubing unclamped. Fill the hydration bladder with water.
5. Place the electronics in a plastic container.

### Results and tips for successful implementation

**Figure 10:**
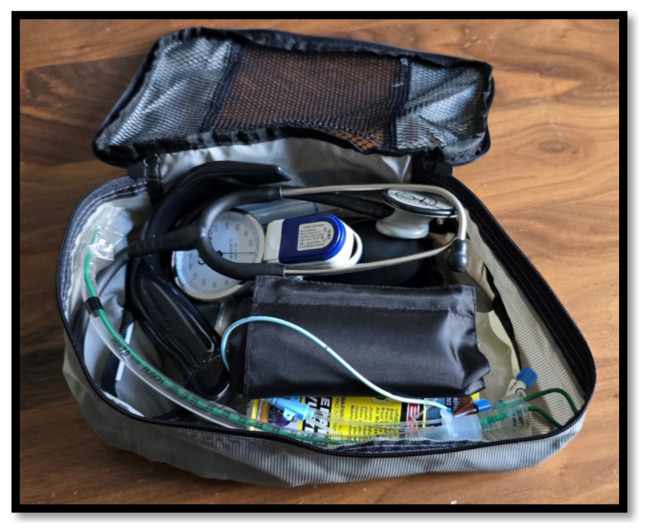
Packing cube containing medical props, such as an endotracheal tube, stethoscope, blood pressure cuff, etc.

*Simulation in a Backpack* is best implemented when traditional simulation equipment, such as a high-fidelity manikin, is not easily accessible. Examples include classrooms, lecture halls, conference rooms, ambulances, homes, and the outdoors. The intent of *Simulation in a Backpack* is to sacrifice some degree of simulation fidelity in exchange for portability, versatility, and speedy set-up. Utilizing packing cubes keeps the materials organized and easy to set up and put away (Figure 10).

### Research Methods

*Simulation in a Backpack* was used as an educational modality for two hours at a time over seven individual sessions from February 2023 until March 2024 at the New York City Poison Center. Each two-hour session included two toxicology-themed emergency medical cases. Learners were primarily resident physicians from Emergency Medicine and Pediatrics. Additional learners included medical students, pharmacists, and pharmacy students. Each session included approximately 25–30 learners for a total of 191.

*Immediately* after each two-hour *Simulation in a Backpack* education session, learners were asked to fill out a twoquestion feedback questionnaire intended for quality improvement. Learners were presented with a Quick Response (QR) code, linking to an anonymous Qualtrics survey. The first question asked for a quantitative feedback instrument, a Net Promotor Score (NPS). The second question asked, “What are your overall thoughts about this training?” and allowed free text entry.

Out of 191 learners, 78 (40.1%) completed a feedback survey. Net Promoter Score (NPS) is an insights tool that can be used to gauge satisfaction with an event and is, in this case, applied to the simulation sessions. NPS scores range from −100 (lowest, everybody is a detractor) to +100 (highest, everybody is a promoter). A positive NPS (i.e. higher than zero) is considered good, an NPS of +50 is excellent, and an NPS above +80 is outstanding. The NPS score for *Simulation in a Backpack* was +81.33.^4^

We also performed a focused qualitative analysis of the written comments using a data abstraction instrument developed for this study. Each author selected “agree” or “disagree” to describe two statements about each comment. We resolved a disagreement over a single comment through mutual discussion. We selected “agree” for 100% (63/63) for the first statement, “This comment reflects a positive learning experience.” We selected “agree” for 4.8% (3/63) for the second statement, “This comment mentions simulation fidelity.”

We believe the learners had a meaningful and engaging educational experience based on the NPS score and written feedback. We further discussed the three comments that mentioned simulation fidelity and agreed that the low-fidelity of the inflatable manikin did not meaningfully degrade the quality of the educational experience. The comments which mention simulation fidelity are listed below verbatim:

- “Love sim. The higher the fidelity the best.”
- “Great scenarios, teaching, and debriefing skills. Although higher fidelity mannequin would add a little bit, I think they key points of the scenario were well taught and came out with the equipment available.”
- “Great! Despite not high fidelity, still got a lot out of it.”

Based on practical experience and feedback from learners, *Simulation in a Backpack* was modified to increase efficiency and decrease set-up time. Given that learners did not appear to have a substantially different learning experience with or without the electronic modules, these were not used in every session. Also, a foot-actuated air-pump decreased the time required to inflate the manikin and was easier to use than a hand-actuated air-pump. Lastly, emphasizing a “fiction contract” wherein the instructor acknowledges the limitations of manikin fidelity allows the learners to engage with the substance of the case more fully.^3^

## Figures and Tables

**Figure 6:**
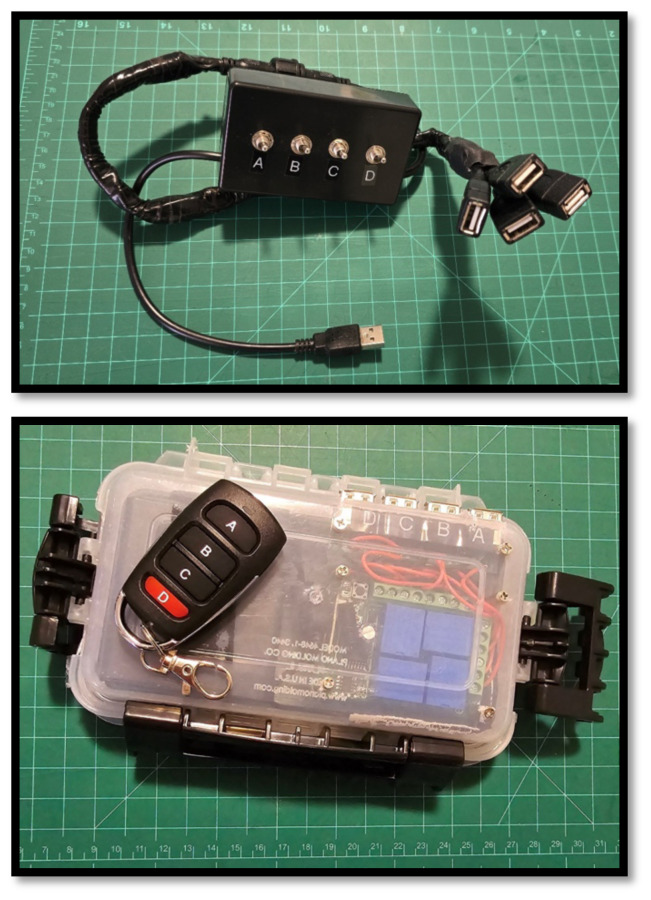
A) Simple and B) radio-frequency controllers for electronic modules. The devices contain four on/off switches which control and distribute the electric supply for each of the four USB outputs. They require a standard 5v USB power source.

**Figure 7:**
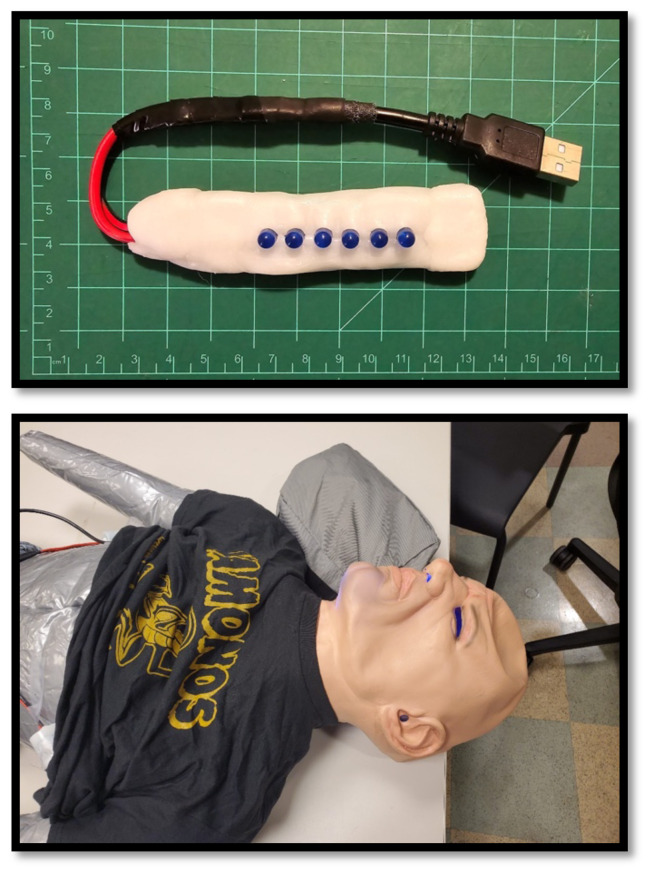
A) Electronic module containing blue LEDs to simulate cyanosis. B) Electronic module containing blue LEDs is placed inside the latex mask. The wire runs underneath the t-shirt to the controller. When activated, this module represents cyanosis. In this image, the blue hue is most notable at the chin and nares.

**Figure 8:**
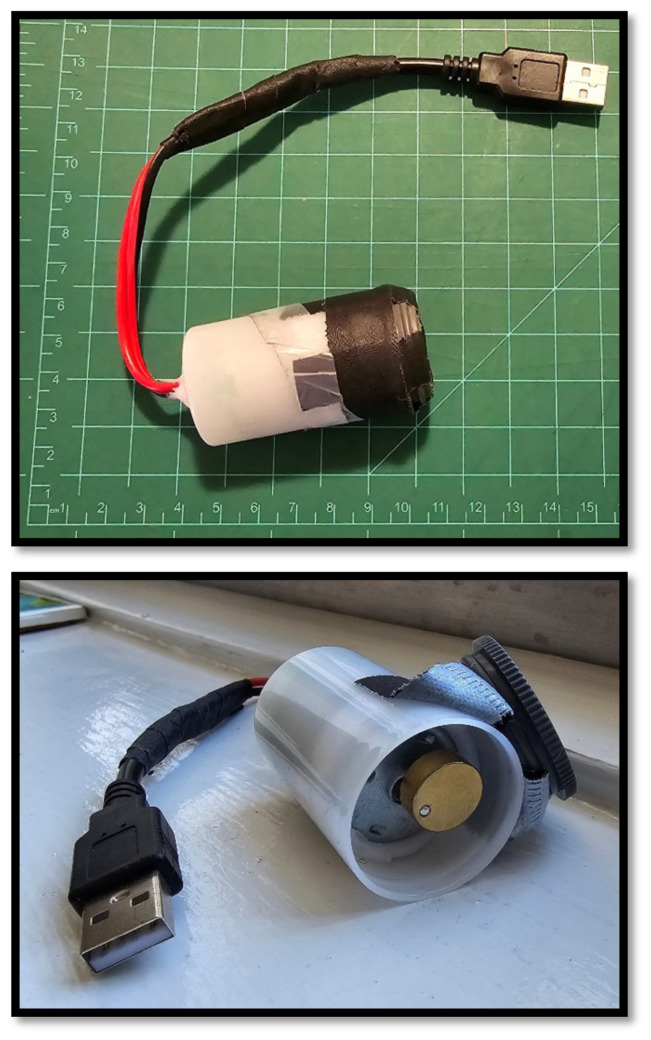
Electronic module vibrating motor to simulate seizures. A) External view. B) View demonstrating the internal vibrating motor.

**Figure 9:**
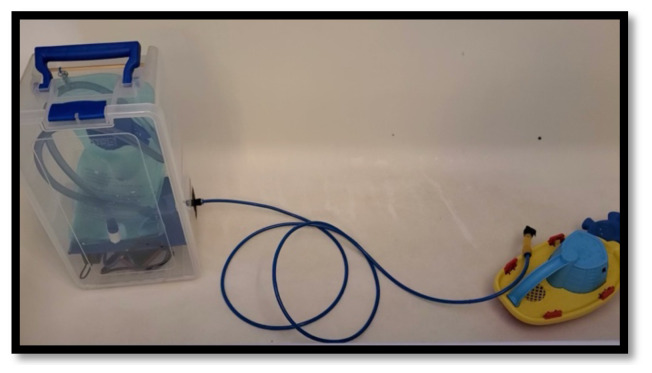
Water pump module. (Left) The plastic housing contains the water bladder, water pump, pulse generator module, and power supply. (Middle) A non-compressible air brake tubing transmits the water to a segment of (Right) latex tubing. A child’s bath toy is provided for scale.
